# Risk factors for subsequent fractures in hip fracture patients: a nested case-control study

**DOI:** 10.1186/s13018-024-04833-6

**Published:** 2024-06-12

**Authors:** Mi Song, Yilin Wang, Yu Jiang, Hongying Pi, Houchen Lyu, Yuan Gao

**Affiliations:** 1 Medical School of Chinese PLA, No. 28, Fuxing Road, Beijing, 100853 People’s Republic of China; 2 Department of Orthopedics, Chinese PLA General Hospital, No. 28, Fuxing Road, Beijing, 100853 People’s Republic of China; 3 Military Health Service Training Center, Chinese PLA General Hospital, No. 28, Fuxing Road, Beijing, 100853 People’s Republic of China; 4 National Clinical Research Center for Orthopedics, Sports Medicine & Rehabilitation, No. 28, Fuxing Road, Beijing, 100853 People’s Republic of China; 5 Department of nursing, Chinese PLA General Hospital, No. 28, Fuxing Road, Beijing, 100853 People’s Republic of China

**Keywords:** Subsequent fracture, Hip fracture, Risk factors, Osteoporosis, Anti-osteoporosis medications

## Abstract

**Background:**

The risk factors for subsequent fractures following an initial hip fracture are not entirely understood. This study examined the clinical characteristics of hip fracture patients to identify potential risk factors associated with a higher risk of experiencing subsequent fractures.

**Methods:**

We conducted a nested case-control study using data from the Chinese PLA General Hospital Hip Fracture Cohort between January 2008 and March 2022. The cases were individuals who experienced subsequent fractures following an initial hip fracture. Each case was matched with up to 2 controls who did not develop subsequent fractures. Important clinical factors were compared across groups, including traditional fracture risk factors and potential risk factors (e.g., comorbidities, falls risk, physical impairment, calcium or vitamin D use, and anti-osteoporosis medications). Conditional logistic regression analyses were used to evaluate the impact of these clinical features as potential risk factors for subsequent fractures.

**Results:**

A total of 96 individuals who suffered from subsequent fractures were matched with 176 controls. The median time between the initial hip fracture and the subsequent fracture was 2.1 years. The overall proportion of patients receiving anti-osteoporosis treatment after initial hip fracture was 25.7%. In the multivariable regression analysis, living in a care facility (OR = 3.78, 95%CI: 1.53–9.34), longer hospital stays (OR = 1.05, 95%CI: 1.00–1.11), and falls after discharge (OR = 7.58, 95%CI: 3.37–17.04) were associated with higher odds of subsequent fractures.

**Conclusions:**

This study showed that living in a care facility, longer hospital stays, and falls after discharge may be independent risk factors for repeat fractures following an initial hip fracture. These findings could be used to identify and manage patients at high risk of subsequent fractures.

**Supplementary Information:**

The online version contains supplementary material available at 10.1186/s13018-024-04833-6.

## Introduction

Hip fractures, affecting an estimated 18% of women and 6% of men, are characterized by substantial morbidity and mortality, and pose a significant health challenge globally [1–4]. Hip fractures are projected to rise to 4.5 million by 2050 [5, 6]. The risk of further fractures following an initial hip fracture is considerably increased [4–10]. Subsequent fractures not only lead to worse clinical prognosis but also place a substantial financial burden on the healthcare system [11–14].

In recent years, there has been increasing awareness and emphasis on preventing subsequent fractures [15]. Clinical guidelines emphasize identifying modifiable risk factors for subsequent fractures [16–18]. While risk factors for initial hip fractures are relatively well-established, our understanding of the determinants for additional fractures remains less clear. Based on the clinical and biological knowledge of fractures, it is reasonable to assume that the risk factors linked to the initial fracture may also be risk factors for subsequent fractures [19, 20]. However, it is necessary to validate the factors within the specific population of hip fracture patients.

Our current knowledge of the risk factors of subsequent fractures is inadequate. A prior Danish population-based study has identified several risk factors for a subsequent hip fracture, including female gender, advanced age, excessive alcohol consumption, living alone, and history of fracture [21]. However, these findings have not been consistently replicated in studies from different settings [22–24]. Most of these previously identified risk factors are non-modifiable [21, 25–27], limiting their utility in the clinical management of hip fractures. More recent studies have expanded the investigation to include modifiable risks, such as comorbidities, exercise, weight management, and anti-osteoporosis medication usage [25, 28, 29, 34]. However, these studies typically focus on baseline variables and neglect post-fracture evaluations [30]. Many of these studies have limitations due to poorly defined subsequent fracture endpoints or incomplete assessment of risk factors, leading to uncertainties in their conclusions [30–33]. Thus, a thorough evaluation of important risk factors, measured during fracture and post-fracture, is needed to understand the risk factors comprehensive.

Although a prospective hip fracture cohort is ideally suited to identifying the risk factors for subsequent fractures, it necessitates the redefinition of potential risk factors and their prospective collection, a time-consuming and resource-intensive process [34, 35]. A nested case-control design enhances efficiency by utilizing pre-collected variables and allowing for the retrospective gathering of additional post-fracture variables of interest, thus offering preliminary evidence in the absence of prospective cohort studies [35].

To examine the risk factors associated with subsequent fractures, we conducted a nested case-control analysis using a large hip fracture cohort.

## Materials and methods

This study followed the Strengthening the Reporting of Observational Studies in Epidemiology (STROBE) reporting guidelines [36].

### Data sources and study cohort

We used data from the Chinese PLA General Hospital Hip Fracture Cohort, a single-center study evaluating the prognosis of hip fracture patients. Patients in the cohort didn’t receive the fracture liaison service or multidisciplinary management. We included patients aged 50 years and above who underwent surgery for an initial hip fracture between January 2008 and March 2022. Patients were excluded if their fracture was not recent (admitted to the hospital more than three weeks after the hip fracture) or if they had missing values for sex and surgery-related data. This study was approved by the Ethics Committee of Chinese PLA General Hospital (No. S2023-059-01).

### Case definition and control selection

We defined cases as individuals who sustained subsequent fractures at different sites following their initial hip fracture. All the subsequent fractures were identified by reviewing both inpatient and outpatient medical charts after their initial hip fractures, as well as conducting telephone reviews post-discharge.

These subsequent fractures encompassed contralateral hip fractures (femoral neck fractures, trochanteric fractures, and subtrochanteric fractures), vertebral fractures, humeral fractures (proximal humerus, shoulder, upper end of the humerus), forearm fractures (forearm, wrist, hand, distal radius), and fractures at other sites. If a patient experienced multiple fractures after initial hip fracture, only the first fracture was considered. We excluded patients with subsequent fractures caused by pathologic conditions, periprosthetic issues, or high-impact trauma.

We adopted a nested case-control study within the cohort to enhance the efficiency. For each case, up to 2 controls (those without subsequent fractures) were matched based on age at the time of initial fracture (within five years), gender, history of fracture, and follow-up time (equal to or exceeding that of the matched case patient) to increase the comparability between groups. Follow-up time was calculated as the duration from the initial hip fracture to the occurrence of subsequent fracture, or March 31, 2022.

### Selection of potential risk factors

Based on current evidence and subject matter knowledge, we considered a wide range of risk factors that could influence subsequent fractures. The baseline predictors were the variables obtained before the discharge of the initial hip fracture. These variables included sociodemographic characteristics (age and sex), lifestyle habits (drinking and smoking), anthropometric measurements (including height, weight and body mass index), malnutrition, comorbid conditions (Alzheimer’s disease, Parkinson’s disease, coronary heart disease, arrhythmia, heart failure, myocardial infarction, valvular heart disease, cerebral infarction, cerebral hemorrhage, encephalopathy sequelae, pneumonia, chronic bronchitis, chronic obstructive pulmonary disease, respiratory failure, anemia, hypertension, type 2 diabetes, chronic kidney injury, eye diseases (glaucoma, cataracts), rheumatic disease, and tumor), fracture type, surgery type (internal fixation, hemiarthroplasty, and total hip arthroplasty), duration of surgery, anesthesia type, hematologic and biochemical tests, and in-hospital postoperative complications (pneumonia, respiratory failure, gastrointestinal bleeding, pulmonary embolism, arrhythmia, angina pectoris, myocardial infarction, heart failure and stroke). Smoking/drinking status was defined as current or not current smoker/drinker [37]. Anemia was defined as hemoglobin less than 130 g/L for men and 120 g/L for women [38], and malnutrition was defined as albumin less than 35 g/L on discharge [39, 40]. Additionally, we calculated the age-adjusted Charlson Comorbidity Index (CCI) [41, 42].

We also included post-baseline predictors as potential risk factors collected after hospital discharge via telephone calls. Follow-up occurred annually after the initial hip fracture. The post-baseline variables of interest included any falls in the following year, level of lower-limb function, extent of physical impairment, use of anti-osteoporosis medications, and calcium or vitamin D supplementation in the most recent follow-up. The lower-limb function impairment was assessed by enquiring whether patients required assistance in performing any of the following activities: walking across a room, getting out of a chair, walking on the level ground outside, and walking up or down stairs [19], the physical impairment was determined by evaluating the use of ambulatory aids, such as a cane or walker [19].

### Statistical analyses

Continuous variables (such as time from admission to surgery, and length of hospital stay) were described as mean with standard deviation and analyzed using *t*-tests. Categorical variables were presented as frequencies with percentages and compared using the Chi-square or Fisher’s exact test. A conditional logistic regression model was used to identify independent predictors for subsequent fractures. We selected variables for the model by considering both clinical significance and statistical relevance. In terms of the statistical criteria, we adopted a significance threshold of *P*-value < 0.1, consistent with previous studies.

We conducted three sensitivity analyses to assess the robustness of our primary findings. First, we sought to determine if the risk factors for composite osteoporotic fractures also applied to hip fractures by restricting cases to those with subsequent hip fractures. Then we considered the impact of age by limiting analysis to patients who were 65 years or older at the time of initial fracture. Last, given the advancements in treatment philosophies, surgical techniques, and medical devices may change over time and ultimately altered the treatment pattern of hip fracture, we restricted patients admitted for their initial hip fracture from 2012 to 2022.

All statistical analyses were performed using R 4.3.0 software (https://cran.rproject.org). Statistical significance was defined as *p*-values < 0.05.

## Results

**Fig. 1:**
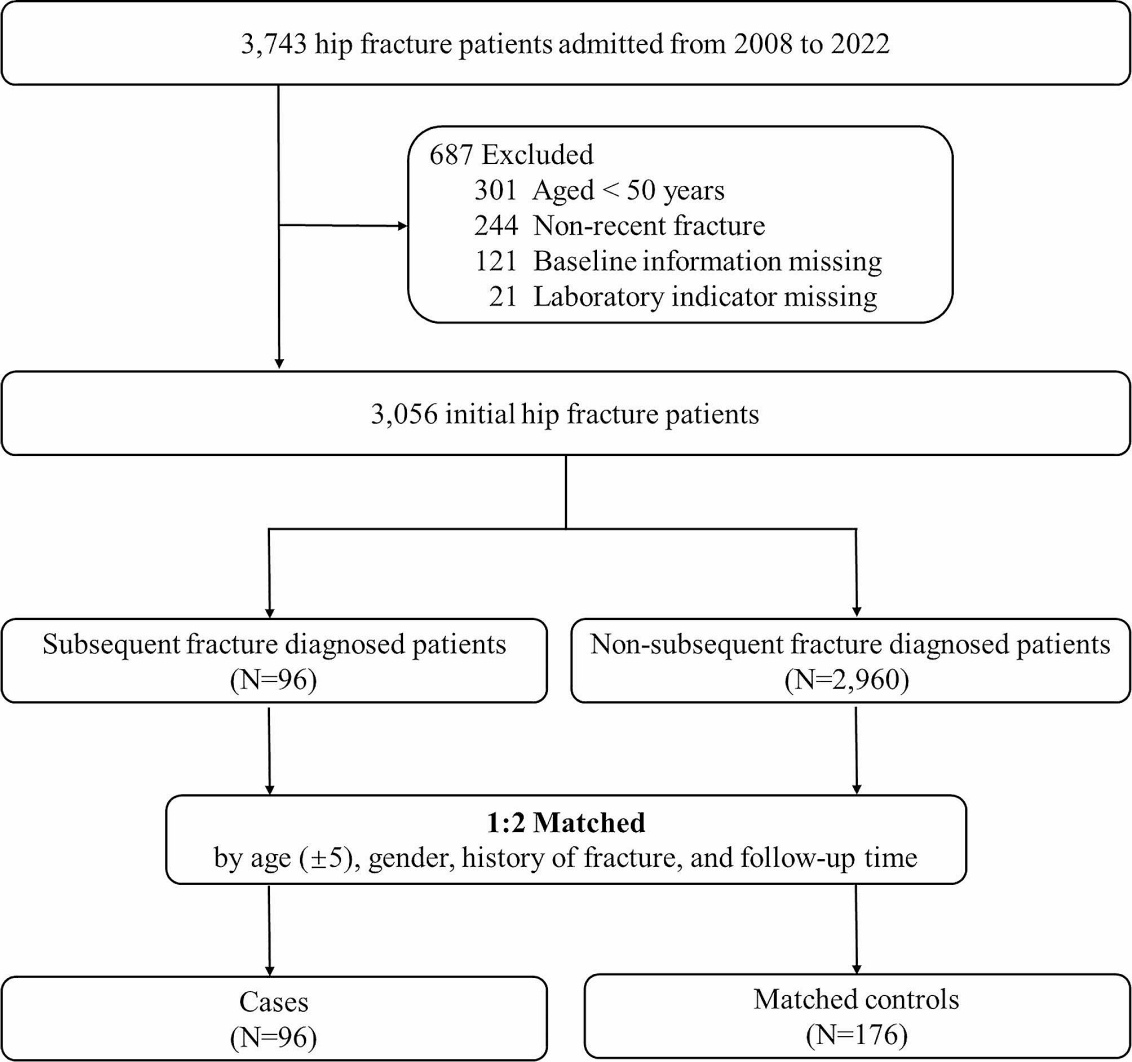
Flow chart

We adopted a nested case-control study design by matching 96 patients who suffered from subsequent fractures with 176 individuals (matching ratio of 1:2) who had a similar age (± 5), gender, history of fracture, and follow-up time after the initial hip fracture but did not experience any subsequent fractures. Thus, this yielded a matched cohort of 272 patients. (Fig. 1 and Supplement Fig. 1).

### Baseline characteristics of the study cohorts

The cases had higher rates of staying in residential care (24.0% vs. controls 10.2%), eye disease (13.5% vs. controls 6.2%), experiencing falls within one year after discharge (38.5% vs. controls 9.7%), having lower-limb function impairment after discharge (49.0% vs. controls 34.1%), having physical impairment after discharge (56.2% vs. controls 36.9%), and having a longer hospital stays (cases 12.86 ± 7.84 vs. controls 11.12 ± 4.58). Additionally, the proportion of patients with a Charlson Comorbidity Index (CCI) score > 3 was higher in the cases group (cases 63.5% vs. controls 60.2%). Only 70 patients (25.7%) in our study used anti-osteoporosis medications after the initial fracture (Table 1).

**Table 1:** Baseline characteristics of the study population

	Cases (*N* = 96)	Controls (*N* = 176)	*P*
**Year of admission**, **No. (%)**			**0.051**
2008–2014	37 (38.5)	91 (51.7)	
2015–2022	59 (61.5)	85 (48.3)	
**Age, No. (%)**			0.874
< 65	13 (13.5)	27 (15.3)	
65–75	25 (26.0)	48 (27.3)	
> 75	58 (60.4)	101 (57.4)	
**Sex**, **No. (%)**			1.000
Male	15 (15.6)	27 (15.3)	
Female	81 (84.4)	149 (84.7)	
**Drinking**	6 (6.2)	8 (4.5)	0.748
**Smoking**	6 (6.2)	13 (7.4)	0.918
**Fracture type**, **No. (%)**			0.722
Intertrochanteric fracture	38 (39.6)	75 (42.6)	
Femoral neck fracture	58 (60.4)	101 (57.4)	
**BMI, No. (%)**			0.491
< 18.5	9 (9.4)	18 (10.2)	
18.5–24	53 (55.2)	84 (47.7)	
> 24	34 (35.4)	74 (42.0)	
**Before initial fracture**, **No. (%)**			
Lower-limb function impairment ^a^	7 (7.3)	12 (6.8)	1.000
Physical impairment	10 (10.4)	13 (7.4)	0.528
Previous fracture	19 (19.8)	24 (13.6)	0.248
Residential care	23 (24.0)	18 (10.2)	**0.004**
Solitude	12 (12.5)	18 (10.2)	0.712
Use of glucocorticoids	4 (4.2)	3 (1.7)	0.409
Use of AOM	3 (3.1)	6 (3.4)	1.000
Use of calcium or VitD	31 (32.3)	32 (18.2)	**0.013**
**Comorbidity**, **No. (%)**			
Neurological disease ^b^	1 (1.0)	8 (4.5)	0.234
Cardiovascular disease ^c^	58 (60.4)	106 (60.2)	1.000
Cerebrovascular disease ^d^	14 (14.6)	19 (10.8)	0.471
Respiratory disease ^e^	4 (4.2)	7 (4.0)	1.000
Hypertension	49 (51.0)	94 (53.4)	0.805
Diabetes	23 (24.0)	38 (21.6)	0.768
Anemia	37 (38.5)	59 (33.5)	0.487
Chronic kidney disease	9 (9.4)	24 (13.6)	0.404
Liver disease	2 (2.1)	5 (2.8)	1.000
Tumor	7 (7.3)	17 (9.7)	0.664
Malnutrition	3 (3.1)	12 (6.8)	0.319
Eye disease	13 (13.5)	11 (6.2)	**0.071**
Hearing impairment	25 (26.0)	34 (19.3)	0.258
Rheumatoid arthritis	6 (6.2)	4 (2.3)	0.184
**Age-adjusted CCI**, **No. (%)**			0.685
≤ 3	35 (36.5)	70 (39.8)	
> 3	61 (63.5)	106 (60.2)	
**Surgery type**, **No. (%)**			0.155
Internal fixation	44 (46.3)	103 (58.5)	
Hemiarthroplasty	37 (38.9)	52 (29.5)	
Total hip arthroplasty	14 (14.7)	21 (11.9)	
**Anesthesia type**, **No. (%)**			0.782
General anesthesia	40 (41.7)	66 (37.5)	
Spinal anesthesia	19 (19.8)	39 (22.2)	
Peripheral nerve block	37 (38.5)	71 (40.3)	
**Duration of surgery, No. (%)**			0.384
< 90 min	29 (30.2)	43 (24.4)	
90–119 min	36 (37.5)	59 (33.5)	
120–150 min	14 (14.6)	39 (22.2)	
> 150 min	17 (17.7)	35 (19.9)	
**Postoperative complications**^**f**^, **No. (%)**	6 (6.2)	7 (4.0)	0.588
**Time from admission to surgery, (SD)**	5.30 (2.85)	5.54 (2.98)	0.945
**Length of hospital stay, mean (SD)**	12.86 (7.84)	11.12 (4.58)	**0.022**
**After initial fracture**, **No. (%)**			
Falls ^g^	37 (38.5)	17 (9.7)	**< 0.001**
Lower-limb function impairment	47 (49.0)	60 (34.1)	**0.023**
Physical impairment	54 (56.2)	65 (36.9)	**0.003**
Use of AOM	26 (27.1)	44 (25.0)	0.818
Use of calcium or VitD	63 (65.6)	79 (44.9)	**0.002**

Notes: Abbreviations: BMI, body mass index; Age-adjusted CCI, the age-adjusted Charlson comorbidity index; VitD, vitamin D; AOM, anti-osteoporosis medications

a. Defined by the need for assistance in performing any of the following activities: walking across a room, getting out of a chair, walking on the level ground outside,

and walking up or down stair

b. Neurological disease includes Alzheimer’s disease and Parkinson’s disease

c. Cardiovascular disease includes coronary heart disease, arrhythmia, heart failure, myocardial infarction, and valvular heart disease

d. Cerebrovascular disease includes cerebral infarction, cerebral hemorrhage, and encephalopathy sequelae

e. Respiratory disease includes pneumonia, chronic bronchitis, chronic obstructive pulmonary disease, and respiratory failure

f. Postoperative complications includes pneumonia, respiratory failure, gastrointestinal bleeding, pulmonary embolism, arrhythmia, angina pectoris, myocardial infarction, heart failure, and stroke

g. Defined as any falls occurring up to 1 year after departure

**Fig. 2:**
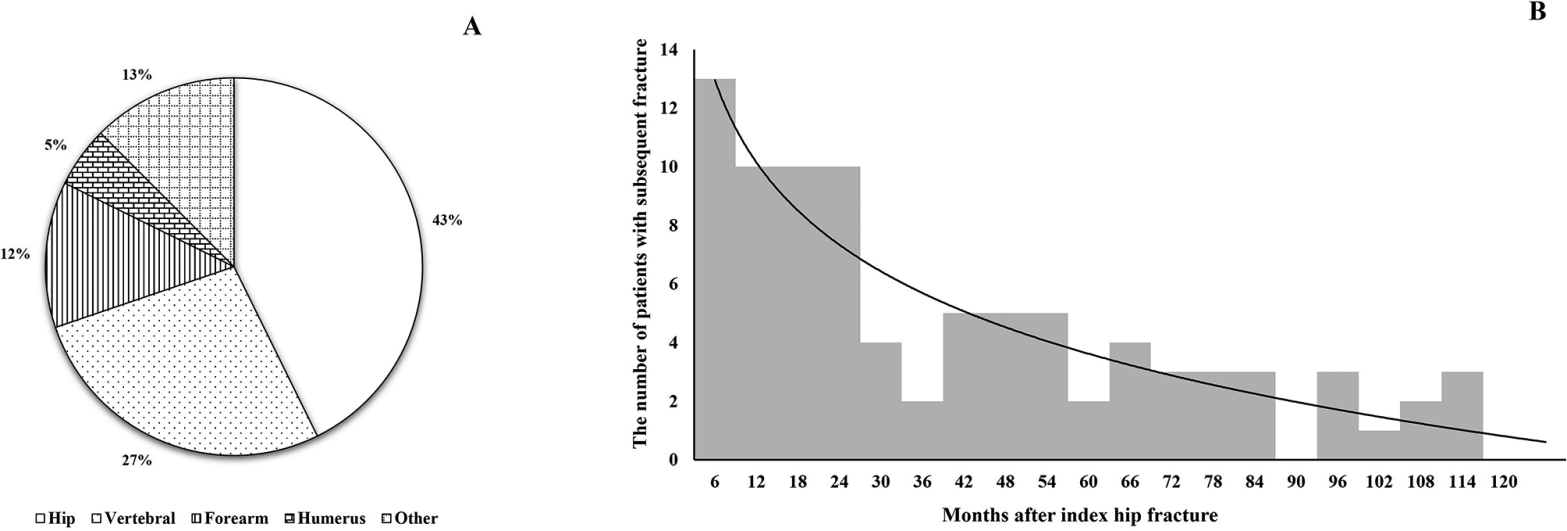
Characteristics of subsequent fractures. **A**. The site of subsequent fractures, **B**. The time course of subsequent fractures

Among the 96 subsequent fracture cases, the hip was the most frequently affected site (*n* = 41, 42.7%). Other common fracture sites were at the vertebral (*n* = 26, 27.1%), forearm (*n* = 12, 12.5%), and humerus (*n* = 5, 5.2%) (Fig. 2A). The median time between the initial hip fractures and the subsequent fractures was 2.1 (range 0.1–12.7) years (Fig. 2B).

### Risk factors of subsequent fractures

In patients with initial hip fractures, the multivariable logistic regression analysis identified several independent risk factors for subsequent fractures including staying in residential care (OR = 3.78; 95% CI, 1.53–9.34), having a longer hospital stay (OR = 1.05; 95% CI, 1.00-1.11) and experiencing falls (OR = 7.58; 95% CI, 3.37–17.04) (Table 2).

**Table 2:** Conditional logistic regression analysis of risk factors on subsequent fracture

	OR (95% CI)	*P*
**Year of admission**		
2008–2014	Reference	
2015–2022	0.81 (0.38–1.72)	0.585
**Age**		
< 65	Reference	
65–75	1.12 (0.42–3.01)	0.825
> 75	0.93 (0.33–2.56)	0.882
**Sex**		
Male	Reference	
Female	1.05 (0.39–2.84)	0.926
**Drinking**	2.38 (0.57–9.99)	0.237
**Smoking**	1.01 (0.31–3.37)	0.982
**BMI**		
18.5–24	Reference	
< 18.5	1.01 (0.34–2.98)	0.982
> 24	0.57 (0.30–1.09)	0.091
**Before initial fracture**		
Previous fracture	0.98 (0.44–2.19)	0.958
Residential care	3.78 (1.53–9.34)	**0.004**
Use of calcium or VitD	1.39 (0.65–2.98)	0.398
**Comorbidity**		
Malnutrition	0.45 (0.11–1.92)	0.281
Eye disease	2.32 (0.81–6.65)	0.117
Hearing impairment	1.70 (0.77–3.73)	0.188
**Age-adjusted CCI**		
≤ 3	Reference	
> 3	0.77 (0.38–1.55)	0.462
**Duration of surgery**		
< 90 min	Reference	
90–119 min	0.67 (0.31–1.44)	0.307
120–150 min	0.53 (0.21–1.34)	0.180
> 150 min	0.75 (0.30–1.84)	0.531
**Length of hospital stay**	1.05 (1.00–1.11)	**0.047**
**After initial fracture**		
Falls ^a^	7.58 (3.37–17.04)	**< 0.001**
Lower-limb function impairment ^b^	0.94 (0.42–2.13)	0.883
Physical impairment	1.78 (0.81–3.87)	0.149
Use of AOM	1.03 (0.49–2.18)	0.935
Use of calcium or VitD	1.56 (0.77–3.16)	0.217

Notes: Abbreviations: BMI, body mass index; Age-adjusted CCI, the age-adjusted Charlson comorbidity index; VitD, vitamin D; AOM, anti-osteoporosis medications

a. Defined as any falls occurring up to 1 year after departure

b. Defined by the need for assistance in performing any of the following activities: walking across a room, getting out of a chair, walking on the level ground outside, and walking up or down stair

### Sensitivity analyses

We conducted a separate analysis that only included controls and subsequent hip fracture cases to assess the significance of risk factors for severe cases. This sensitivity analysis reaffirmed that residence in a care facility (OR = 3.53; 95% CI, 1.02–12.20), and experiencing falls within a year (OR = 6.49; 95% CI, 2.22–18.94) after initial fracture were significantly associated with subsequent fractures (Table 3).

**Table 3:** Sensitive analysis of the risk factors for subsequent fracture

	Sensitivity analysis ^1^ (*N* = 217)			Sensitivity analysis ^2^ (*N* = 232)			Sensitivity analysis ^3^ (*N* = 225)	
	OR (95% CI)	*P*		OR (95% CI)	*P*		OR (95% CI)	*P*
**Year of admission**								
2008–2014	Reference			Reference			Reference	
2015–2022	0.55 (0.19–1.59)	0.269		0.80 (0.36–1.81)	0.593		1.78 (0.72–4.37)	0.209
**Age**								
< 65	Reference			––			Reference	
65–75	1.02 (0.19–5.49)	0.983		Reference			1.52 (0.47–4.97)	0.485
> 75	2.70 (0.56–13.09)	0.218		0.82 (0.35–1.92)	0.653		0.65 (0.19–2.17)	0.481
**Sex**								
Male	Reference			Reference			Reference	
Female	0.97 (0.26–3.57)	0.963		0.71 (0.22–2.27)	0.569		0.97 (0.31–3.07)	0.959
**Drinking**	3.32 (0.49–22.64)	0.220		1.06 (0.15–7.49)	0.950		3.92 (0.76–20.12)	0.102
**Smoking**	2.05 (0.41–10.26)	0.382		1.04 (0.27–4.04)	0.958		0.68 (0.15–3.02)	0.616
**BMI**								
18.5–24	Reference			Reference			Reference	
< 18.5	0.79 (0.16–3.86)	0.772		0.84 (0.26–2.72)	0.777		1.08 (0.35–3.34)	0.895
> 24	0.50 (0.21–1.22)	0.130		0.56 (0.27–1.15)	0.115		0.44 (0.20–0.95)	**0.037**
**Before initial fracture**								
Previous fracture	0.88 (0.28–2.75)	0.823		0.80 (0.31–2.08)	0.651		0.81 (0.33–2.01)	0.649
Residential care	3.53 (1.02–12.20)	**0.046**		7.59 (2.50–23.06)	**< 0.001**		3.49 (1.34–9.09)	**0.011**
Use of calcium or VitD	2.33 (0.80–6.81)	0.123		1.19 (0.51–2.77)	0.679		1.21 (0.52–2.81)	0.650
**Comorbidity**								
Malnutrition	0.47 (0.07–2.94)	0.419		0.21 (0.04–1.23)	0.083		0.81 (0.18–3.64)	0.780
Eye disease	2.10 (0.51–8.62)	0.303		3.34 (1.07–10.44)	**0.038**		1.91 (0.61–5.99)	0.269
Hearing impairment	1.64 (0.57–4.74)	0.357		1.59 (0.68–3.72)	0.283		1.67 (0.65–4.28)	0.288
**Age**–**adjusted CCI**								
≤ 3	Reference			Reference			Reference	
> 3	0.44 (0.16–1.21)	0.113		0.87 (0.40–1.89)	0.729		0.93 (0.41–2.14)	0.865
**Duration of surgery**								
< 90 min	Reference			Reference			Reference	
90–119 min	1.11 (0.37–3.33)	0.851		0.68 (0.30–1.55)	0.356		0.65 (0.27–1.56)	0.337
120–150 min	0.78 (0.21–2.87)	0.707		0.46 (0.17–1.26)	0.130		0.49 (0.17–1.43)	0.193
> 150 min	1.61 (0.47–5.52)	0.449		0.97 (0.37–2.57)	0.953		0.69 (0.24–1.96)	0.481
**Length of hospital stay**	1.03 (0.95–1.11)	0.511		1.04 (0.97–1.11)	0.316		1.07 (1.01–1.14)	**0.021**
**After initial fracture**								
Falls ^a^	6.49 (2.22–18.94)	**< 0.001**		8.57 (3.47–21.21)	**< 0.001**		6.94 (2.98–16.15)	**< 0.001**
Lower-limb function impairment ^b^	0.95 (0.31–2.94)	0.930		0.91 (0.38–2.19)	0.830		1.19 (0.46–3.05)	0.718
Physical impairment	3.71 (1.19–11.52)	**0.023**		1.64 (0.70–3.84)	0.250		1.84 (0.74–4.58)	0.189
Use of AOM	0.50 (0.16–1.54)	0.229		1.28 (0.55–2.94)	0.565		1.08 (0.45–2.57)	0.868
Use of calcium or VitD	1.11 (0.42–2.89)	0.837		1.14 (0.52–2.49)	0.739		1.78 (0.80–3.99)	0.159

Notes: Abbreviations: BMI, body mass index; Age–adjusted CCI, the age–adjusted Charlson comorbidity index; VitD, vitamin D; AOM, anti–osteoporosis medications

a. Defined as any accidental falls occurring up to 1 year after departure

b. Defined by the need for assistance in performing any of the following activities: walking across a room, getting out of a chair, walking on the level ground outside, and walking up or down stair

1. Sensitivity analysis by restricting patients only to those who suffered subsequent hip fracture

2. Sensitivity analysis by including patients who were 65 years or older at the time of initial fracture

3. Sensitivity analysis by including patients who were admitted for their initial hip fracture from 2012 to 2022

To account for the impact of age and the long duration of our study, we further analyzed risk factors in specific subgroups: patients aged ≥ 65 years, and patients restricted to the recent ten years (from 2012 to 2022). Results of sensitivity analyses were consistent with the primary analysis, that is, residence in a care facility, longer hospital stays and experiencing falls within a year after discharge were significantly associated with increased odds of subsequent fractures (Table 3).

## Discussion

### Main findings

This study identified several risk factors associated with subsequent fractures after the initial hip fracture including staying in residential care, longer hospital stays, and accidental falls following the initial hip fracture.

### Comparison with existing literature

Risk factors for subsequent fractures in individuals who have experienced an initial fracture carry significant clinical implications. However, high-quality evidence remains insufficient. The study of risk factors for subsequent fractures necessitates the implementation of cohort designs that include a population of individuals with prior fractures. This can be accomplished through prospective cohort designs, national claim databases, or electronic medical records. However, prospective cohort studies require long-term follow-up. Several large fracture registries are yet to publish results on the risk factors associated with subsequent fractures [7, 43–45]. National registries and electronic medical records are limited by their ability to define the occurrence of subsequent fractures accurately [21, 22]. Although studies based on numerous databases report on the trend of subsequent fractures, they fall short of examining the risk factors associated with these sequential fractures. Without evidence from cohort studies investigating subsequent fractures, we adopted a nested case-control design to screen for potential risk factors contributing to subsequent fractures.

Accidental falls are strongly associated with fractures and increase the risk of subsequent fractures by over 20 times (OR, 6.67–22.52) [29, 46]. Our study observed a similar association between accidental falls and subsequent fractures, with an OR of 7.58 (95% CI, 3.37–17.04). This association was consistent among sensitivity analyses, including restricted the study population to those experienced subsequent hip fractures (OR = 6.49; 95% CI, 2.22–18.94), to those who were 65 years or older (OR = 8.57; 95% CI, 3.47–21.21), and to patients who were admitted for their initial hip fracture from 2012 to 2022 (OR = 6.94; 95% CI, 2.98–16.15). Given that 95% of hip fractures result from accidental falls [47–50]. There is an urgent need to incorporate fall prevention measures in the management of hip fracture patients to reduce the occurrence of subsequent fractures.

Individuals residing in nursing homes are reported to have a significantly higher risk of hip fractures compared to those living in the community [51, 52]. Consistent with these findings, our study revealed that patients living in nursing homes were 3.78 times more likely to experience subsequent fractures than community-based individuals. Patients residing in these nursing care institutions were older, female, and had impaired ambulation [53], all of which contribute to an increased risk of falls and potentially result in worse functional outcomes [54]. As China continues to witness an increase in the proportion of the aging population, there will be a growing number of elderly individuals residing in nursing homes. It is imperative to prioritize and enhance health management strategies for this specific and vulnerable population.

We found an increased risk of subsequent fractures among patients aged 65 years or older with eye disease. An extensive nationwide population-based study involving 87,415 hip fracture patients found that patients with eye disease were three times more likely to suffer from a recurrent hip fracture [21]. Similarly, a previous meta-analysis assessed eye disease to be linked to a greater incidence of recurrent hip fracture (OR 2.09; 95% CI, 1.06–4.12) [55], a plausible observation since eye disease is a risk factor for accidental falls [47]. Therefore, healthcare providers should actively screen and refer hip fracture patients with eye disease for specialized eye care and regular monitoring if a multidisciplinary team is not available.

Individuals with better functional status are more likely to experience subsequent fractures [33, 56]. This observation that may be attributed to independent mobility among these patients, which can lead to higher levels of physical activity and consequently increase the risk of falls. Interestingly, our study provides contrasting results whereby patients with limited mobility had an increased risk of subsequent hip fractures (OR = 3.71; 95% CI, 1.19–11.52). The findings of our study can be rationalized from the perspective that poor physical function may often lead to inactivity and immobility that may cause muscle atrophy [57] and ultimately increase the risk of falls. It is important to note that our study provides conservative estimates since, in our analysis, we excluded patients with severely compromised functional status and those who may have become bedridden or died following the initial hip fracture [3].

Prophylactic treatment against osteoporosis is recommended to reduce the likelihood of subsequent fractures, particularly in high-risk populations [28]. Both guidelines and trials emphasize the use of calcium and vitamin D in conjunction with anti-osteoporosis medications [18, 58]. However, the multivariable regression analysis showed no statistically significant difference in anti-osteoporosis medications, vitamin D or calcium supplements between the two groups. This could possibly be explained by the small sample size and low rate of anti-osteoporosis medications use, which limited the power of the study. We also found that the intake of these medications among patients with initial hip fractures is low, accounting for only 25.7% in our study, emphasizing a significant treatment gap for patients suffering from hip fractures. A similar pattern emerged from other studies including a prospective study involving ten countries, which showed less than 20% of women with new fractures received anti-osteoporosis medications within one year [59]. In yet another study involving a review of healthcare data from 15 countries, the proportion of use of osteoporosis treatment varies from 12.9 to 50.3% [3]. The reasons for the phenomenon could be an underestimation of osteoporosis severity by both healthcare providers and patients, as well as concerns regarding the efficacy and potential side effects of therapeutic medications [60]. Therefore, it is necessary to advocate for enhanced patient education and improved communication between healthcare providers and patients to ensure better initiation of anti-osteoporosis treatment.

Our sensitivity analyses supported the main findings, indicating robust results. Owing to concerns about the poor baseline health status and prognosis of relatively severe patients (subsequent hip fracture patients and older patients), we hypothesize that the risk factors of subsequent fractures in these patients might differ from the general hip fracture patients. However, the sensitivity analysis results did not support our hypothesis. Considering the potential influence of admission time, we postulated patients’ admission time could have altered the factors influencing subsequent fractures. Nevertheless, the research outcomes remained incongruent with our assumptions. These sensitivity analyses suggest the identified risk factors for subsequent fractures in this study are likely to be widely applicable.

### Strengthens and limitations

This study’s major strength is its design, whereby we comprehensively evaluated a series of potential risk factors, including those present during the hospitalization and post-discharge periods. While yielding vital insights, we acknowledge some limitations to our study. First, as a single-center study, the generalizability of the results to another population may be limited. Second, follow-up data after hospital discharge were obtained via telephone calls, which may suffer from recall bias. Third, the occurrence of subsequent fractures may be underestimated since most vertebral fractures are asymptomatic. Fourth, we did not include bone mineral density as a variable in our analysis due to the low rate of bone mineral density testing, only 17.6% of the patients had bone mineral density data available. Future prospective studies with bone mineral density data are needed to validate these findings. Fifth, we could not consider the dosage, duration of treatment, and compliance in using anti-osteoporosis medications due to the underuse of anti-osteoporosis medications. Last, we did not have detailed information on some potential risk factors, such as smoking, drinking, and baseline functional status, preventing us from assessing their association with subsequent fractures.

### Further research

Due to the limited number of patients and the retrospective nature of the study, caution should be applied when interpreting the results of the current study. Our preliminary findings invite confirmatory prospective studies involving larger sample sizes, multicenter design, and longer follow-up periods. Additionally, further research is required to examine the role of bone mineral density, dose, duration, and compliance to prophylactic osteoporosis treatment in the developing subsequent fractures.

### Clinical implication

Considering the consequences of subsequent fractures, we strongly recommend early risk evaluation and appropriate interventions to minimize the associated risks. Physicians need to acknowledge the treatment gap (low rate of osteoporosis pharmacotherapy) in osteoporosis management and strategies must be developed and implemented to enhance the timely initiation of anti-osteoporosis medications following hip fractures. The findings of our study hold significant clinical implications for the early identification and appropriate interventions among high-risk patients.

## Conclusion

Our study provides evidence that residing in a nursing care facility, longer hospital stays, and accidental falls are associated with increased odds of subsequent fractures. We identified an alarming gap in anti-osteoporosis treatment among hip fracture patients and recommended that these findings be used to identify and manage patients at high risk of subsequent fractures.

### Electronic supplementary material

Below is the link to the electronic supplementary material.

Supplementary Material 1

## Data Availability

No datasets were generated or analysed during the current study.

## Abbreviations

BMI: Body mass index
Age-adjusted CCI: The age-adjusted Charlson comorbidity index
VitD: Vitamin D
AOM: Anti-osteoporosis medications
