# Supplementary figures and images for: Risk factors for subsequent fractures in hip fracture patients: a nested case-control study
Source: J Orthop Surg Res. 2024 Jun 12;19:348. doi: 10.1186/s13018-024-04833-6 (PMC11167847; doi:10.1186/s13018-024-04833-6)

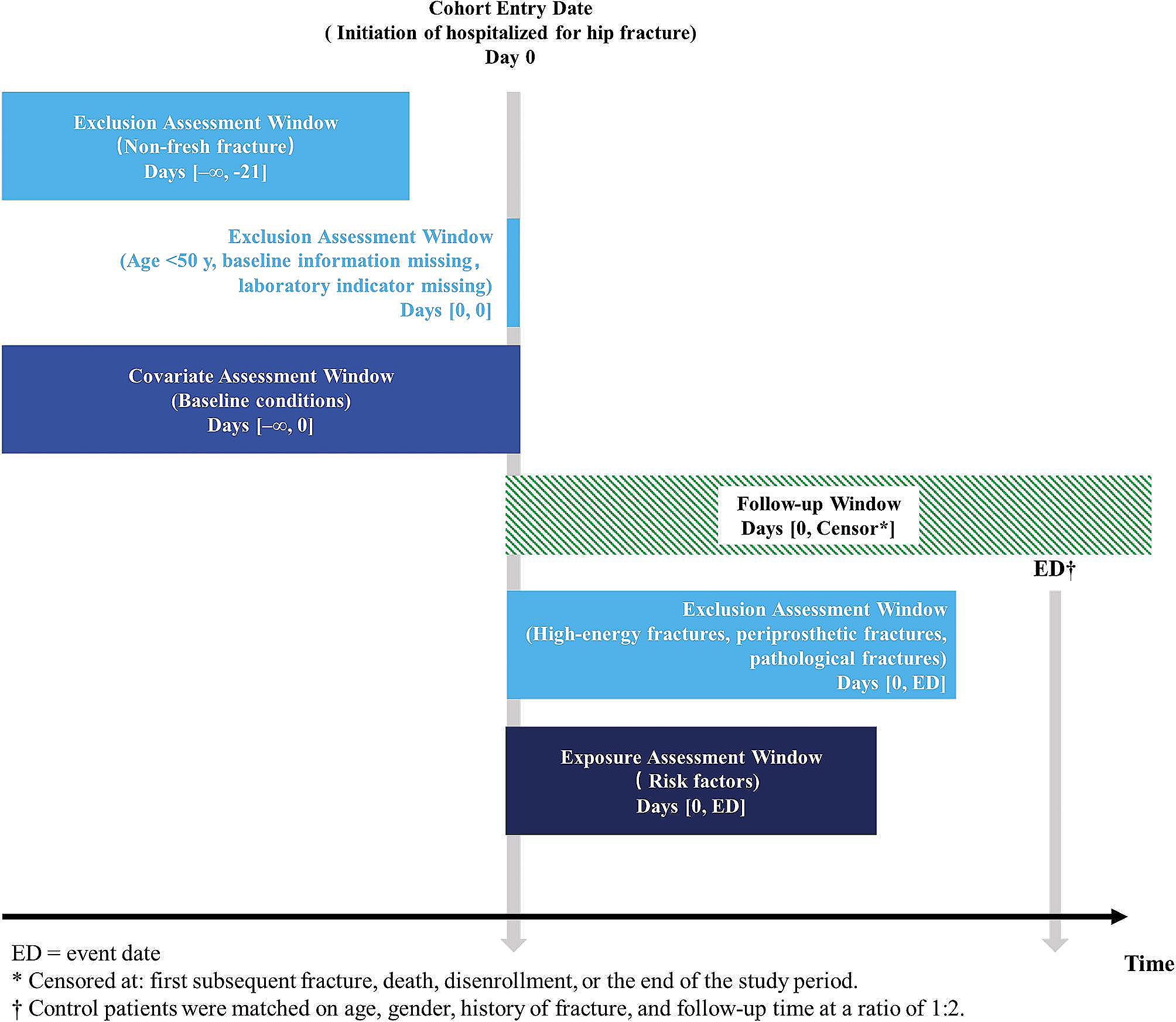

Supplement: Supplementary file 1 — Supplementary Material 1 [file 13018_2024_4833_MOESM1_ESM.png]
